# Dynamic stability of the financial monitoring system: Intellectual analysis

**DOI:** 10.1371/journal.pone.0276533

**Published:** 2023-01-20

**Authors:** Olha Kuzmenko, Yuriy Bilan, Evgenia Bondarenko, Beata Gavurova, Hanna Yarovenko

**Affiliations:** 1 Economic Cybernetics Department, Institute of Business, Economics and Management, Sumy State University, Sumy, Ukraine; 2 Faculty of Economics and Management, Czech University of Life Sciences Prague, Prague, Czech Republic; 3 Department of Financial Technologies and Entrepreneurship, Institute of Business, Economics and Management, Sumy State University, Sumy, Ukraine; 4 Faculty of Management and Economics Tomas Bata University in Zlin, Zlín, Czech Republic; Szechenyi Istvan University: Szechenyi Istvan Egyetem, HUNGARY

## Abstract

**Introduction:**

Although there is a growing number of scientific publications on financial monitoring, combating money laundering, the shadow economy, and the impact of corruption on economic development, further research needs to determine the stability of the national financial system in dynamics. The dynamic stability of the national financial monitoring system subjects will allow to adequately assess the effectiveness of the existing national financial monitoring system in each country and determine the influential factors.

**Materials and methods:**

The article investigates an approach to identifying the dynamic stability of the national financial monitoring system subjects based on the calculation of the integrated indicator of the country’s financial system propensity to ALM, vector autoregression (VAR) model taking into account time lag. The proposed integrated indicator allowed to adequately assess the existing financial monitoring systems of the countries (15 countries of the European Union for 2000–2020: Austria, Belgium, Cyprus, Estonia, Finland, France, Greece, Ireland, Italy, Latvia, Malta, Netherlands, Portugal, Slovak Republic, Spain). In addition, vector autoregression models (VAR) of the dependence of the country’s financial system propensity to ALM on the regressors Government Integrity, Index of economic freedom, Monetary Sector credit to the private sector (% GDP), were built, taking into account time lags in general and for each studied country.

**Results:**

According to the modeling results, the national financial monitoring systems in Austria, Belgium, Estonia, Finland, France, Ireland, Netherlands, Slovak Republic, Spain were resistant to money laundering. It is vice versa in Malta, Greece, Cyprus, Portugal, Italy, Latvia. These conclusions are also confirmed based on a binary approach. Such exogenous variables as Government Integrity (with a lag of 2 years) and the Index of economic freedom (taking into account the time delays of the regression reflection under the influence of this regressor for 1 and 2 years) have a statistically significant effect on the country’s financial system.

**Conclusion:**

The general vector autoregression (VAR) model shows that the current value of the country’s financial system propensity to ALM by 92.78% is determined by its previous value. With an increase of Government Integrity by 1%, the country’s financial system’s propensity to ALM will decrease by 0.000616 units with a lag of two years. The nature of the impact made by the Index of economic freedom on the performance feature was specific—when this indicator increases by 1% for a lag delay in one year, the PFSALM value will decrease by 0.001997 units, and for a lag delay of two years it will change the trend and increase by 0.003076 units per unit, respectively.

## Introduction

Global transformation processes, the extension of global economic ties, and the introduction of new technologies in the financial sphere significantly increase the overall vulnerability of the international financial system and, as a result, pose a threat to each country’s national security. One of the major threats to any national economy is the problem of Anti-Money Laundering (AML).

Such international organizations as the United Nations, the Council of Europe, the World Bank, the International Monetary Fund, the Financial Action Task Force (FATF) focus their attention on AML.

The problem of creating an integrated financial monitoring system is important and relevant today; its solution can contribute to the country’s socio-economic development, reform the budget and social security system, the efficient use of state property and ensure an adequate national security.

Within the framework of financial activity, financial monitoring is a financial and legal instrument of anti-money laundering and anti-terrorist financing.

The need to consider financial monitoring as a mandatory condition for ensuring financial security is justified by the negative consequences that the state and society suffer from the state’s money laundering and terrorist financing. Money laundering threatens the state’s national security since it attracts organized criminal groups to the country, promotes the creation of "home-grown" illegal communities, increases the overall crime rate, and, as a result, forces the state to increase law enforcement. Thus, the creation of a financial ground for criminal activity entails an increase in government expenditure on law enforcement, which usually has a negative impact on government funding of investment projects or capital expenditures, slows GDP growth, increases the budget deficit.

It is important to note that money laundering is particularly detrimental to emerging markets since money laundering becomes the financial basis for organizing and carrying out armed coups, corruption, and establishing the financial dependence of legitimate authorities on large criminal groups within the country.

Uncontrolled movement of significant financial resources—illegal (unaccounted) criminal proceeds in a short period is also a serious threat to financial security.

Criminal proceeds, which in the short term may benefit economies in need of investment, including foreign, can undermine the national economy of any state since it is impossible to predict and plan the activities of entities engaged in money laundering or other property.

The unpredictability of money launderers in financial markets leads to sharp and significant fluctuations in demand for financial resources, changes in exchange and interest rates, and as a result, potential omissions in the financial policy of public financial authorities that assess capital markets, which can lead to financial difficulties in the state and economic entities, up to the bankruptcy of the latter.

That is why the state authorities in most countries make significant efforts to create and ensure the effective functioning of the national financial monitoring systems of their national economies. An important peculiarity of the financial monitoring system is its resilience to various economic entities’ actions (including criminal ones) and other external factors. The financial monitoring system is also considered stable, effectively countering money laundering even with a significant change in the external environment parameters. The necessity to determine the sustainability of financial monitoring systems in European countries and the factors that have a key impact on such sustainability identified the chosen research topic.

## Theoretical framework

In recent decades, the issue of financial monitoring in each country has been very relevant. The growing attention to the problem of improving the financial monitoring effectiveness among scientists and practitioners is largely caused by its importance for ensuring the state’s economic security. In addition, the urgency of enhancing financial monitoring systems increases with the further development of financial and payment technologies. Thus, it leads to an increase in the number of scientific papers in this field around the world.

It is advisable to analyze existing scientific papers and practical recommendations on financial monitoring and related industries to understand the research problem better.

In their study, scientists Leonov, S., Yarovenko, H., Boiko, A., & Dotsenko, T. [1] developed an information system prototype for intrabank monitoring of money laundering transactions. Since banks are the key subjects of financial monitoring in many countries, it is reasonable to pay considerable attention to the automation of these systems in banking institutions when developing an effective anti-money laundering system. According to scientists, this automation will increase bank efficiency by studying all banking operations without exception, leveling the human factor, maximizing the detection speed of suspicious transactions and minimizing losses related to the payment of penalties imposed by regulatory authorities. It is established that the information system prototype for monitoring operations related to money laundering through banks should consist of a model of monitoring business processes in an automated system environment, DFD-model of automatic monitoring of banking operations, structural database model, user interface forms and include the logic of checking business rules.

Subeh, M.A., Boiko, A. [2] presented a study on building a scientific and methodological approach to assessing the State Financial Monitoring Service effectiveness as part of a national anti-money laundering or anti-terrorism financing system in Ukraine. The scientific and methodological approach proposed by the authors allowed us to conclude that the State Financial Monitoring Service in Ukraine works inefficiently. In addition, the efficiency of its work has a negative tendency to decrease. Scientists identified that the reasons are low integrated index, low efficiency of state authorities’ activities (described by the input flow), low efficiency of taken measures (represented by the services), and inefficiency of results obtained from applied steps (described by the flow of output). One should also note that the developed scientific and methodological approach is universal and can be used to assess the effectiveness of any government agency or commercial entity the activities of which are related to three components: input information, its service, and conclusions in the form of a flow of results.

In the process of studying financial monitoring, Alibeki H., Samsonov M. [3] paid considerable attention to the study of banking supervision "off-site", namely, remote monitoring of banks. The authors have identified consolidated supervision and systematic stress testing by banks as effective methods of remote banking monitoring. According to scientists, an effective stress testing system will provide a comprehensive, integrated, and promising set of measures for a banking organization to help identify and measure its material risks and vulnerabilities. The stress testing methodology of each specific banking institution should be developed in proportion to its size, complexity, business activity, and overall risk profile. In this context Belas et al. [4] emphasize bank models are not perfect and give quite unreliable results, respectively; they contribute to the procyclical tendencies of the financial system.

In their work, Bukhtiarova et al. [5] investigated evaluating the effectiveness of financial monitoring measures in Ukraine. This problem is especially relevant for Ukraine since the country has a bank-centric financial market model (about 90% of assets pass through the banking system). According to official data, 50% of economic activity in Ukraine ends with money laundering. The article presents an improved method that quantifies the efficiency of the financial monitoring system in commercial banks of Ukraine based on the calculations of the integrated index. The index indicates the protection degree dynamics of the financial system from the threat of money laundering based on the feasibility and effectiveness of financial monitoring in the banking system. The method proposed by the authors can be used to assess the effectiveness of the financial monitoring system in any country to improve the anti-money laundering system through the banking system.

Modern scientists in the study of financial monitoring pay considerable attention to the shadow economy as one of the most important factors, which leads to an increase in the number of money laundering cases. According Ginevicius, et al. [6] the higher the level of national economic development, the lower the size of the shadow economy. The long-run analysis revealed that shadow economies negatively affected foreign direct investment inflows (Bayar et al. 2020).

For example, Zolkover, A., Terziev, V. [7] analyzed research areas related to the shadow economy. The authors used VOSviewer, Scopus, and Web of Science (WoS) for the analysis. The authors conducted a study based on 5361 work of the Scopus database and 3773 articles of the Web of Science. Time analysis has shown that in 2014–2015, the number of articles on the shadow economy began to increase. At the same time, the research focal point has moved from general issues (shadow sector assessment, impact on the labor market, etc.) to the problem of transition from the informal to the formal economy. In 2019, the number of works on the shadow economy increased by 95% compared to 2014, according to the Scopus database—by 29%. Most articles with the keyword “shadow” (informal, hidden, etc.) economy were published in the following subject area, according to Scopus: social sciences; economics, econometrics, and finance; business, management and accounting; ecology; arts and humanities, and according to WoS: business economics; sociology; governance; state law; development research; other topics of social sciences; environmental sciences; territorial research. Most articles on the shadow economy have been published by scientists from the United States, Britain, India, Germany, and South Africa. These results prove that the informal economy and its transition to a formal one corresponds to the current trends of modern regulation.

In their study, Bilan, et al. [8] considered how the size of the shadow economy of European countries affects the GDP carbon intensity of the economy. As a result of calculations (confirmation of stationarity (Dickey-Fuller test), cointegration of data series (Johansen test), equation of cointegration of dependence of GDP carbon intensity on the shadow economy, it was confirmed that European countries are characterized by increasing GDP carbon intensity of economy with increasing the shadow economy.

Lyulyov, et al. [9] analyzed the driving factors for the shadow economy development in transition economies. It was found that an increase in GDP per capita in the selected countries with transition economies by 10% reduces the shadow economy by 1.2%; an increase in foreign direct investment by 10% reduces the shadow economy by 0.5%; a 10% improvement in energy efficiency correlates with a 2% growth in the shadow economy. It was also found that increasing the tax rate by 10% raises the shadow economy by 1%. Based on the results, the authors formed key policy vectors for countries with transition economies that will reduce the shadow economy: stimulate economic growth and reform the structure of the tax system towards a larger share of indirect taxes.

In their study, Zolkover, A., Georgiev, M. [10] summarized the arguments and counterarguments in the scientific discussion on the problem of combating shadow activity in terms of macroeconomic stability. The authors focused on determining the allowable level of investment transactions with fictitiousness, which correspond to the balance between the shadowing of the national economy and its macroeconomic stability. The relevance of this scientific problem is that the shadow investment activity distorts the market mechanism and makes it impossible to attract financial resources for expanded reproduction in the country. The calculations performed by the authors proved that there is a nonlinear functional dependence of the investment operations with fictitiousness features on the level of shadowing of the national economy and its macroeconomic stability.

Shpak et al. [11] analyzed the shadow industry in the Ukrainian regions, assessing the integrated indicator of financial and economic security of financial and economic security the industry. The study’s key results were: 1) development of theoretical and applied approaches to the impact of the shadow economy on public administration for the financial and economic security of industry in the regions; 2) improving the methodology of public policy analysis regarding the shadow economy in this area. Recommendations on state policy measures to reduce the shadow industry level in the regions were also presented. In addition, based on the analysis, a matrix of strategic zones "The level of the shadow economy—the level of financial and economic security", which can be used for public administration decisions depending on the strategic area in which the region is formed.

In addition to the shadow economy, in terms of the study of financial monitoring, scholars worldwide also pay attention to corruption, given that corruption contributes to the development of the shadow economy and increases the number of money laundering transactions. The study of Nguyen, T.A.N., Luong, T.T.H. [12], where the authors examined how corruption and the shadow economy interact with economic growth in 17 selected Asian countries, is particularly interesting. This paper analyzes the annual data of the World Bank, Transparency International, and the International Monetary Fund for the period 2000–2015 to assess whether corruption and the shadow economy affect economic growth. The authors’ calculations show that the corruption index has a statistically significant and positive impact on economic growth, while the shadow economy has a significant negative effect. In addition, reducing the size of the shadow economy may be more beneficial for emerging markets in terms of economic development opportunities. The results also indicate that in these countries, foreign direct investment, government spending, tax revenues and inflation have a positive effect on growth, while remittances do not have a significant impact on the economy.

Simovic M. [13] conducted a similar study for Eastern European countries (Montenegro, Bosnia and Herzegovina and Serbia). The subject of this study is the relationship between corruption and economic growth in the three selected countries. The starting point of the study was the hypothesis that corruption has a significant negative impact on economic growth in Southeast Europe. The author used the Corruption Perceptions Index and the gross domestic product (GDP) per capita for the analysis. The study period starts from the beginning of 2003 to the end of 2018. The results of the study confirmed the initial hypothesis of explaining the corruption impact on economic growth in the countries of South-Eastern Europe.

Brychko et al.(2021) [14] conducted a study of the mediating impact of the crisis of confidence in the financial sector on macroeconomic stability indicators due to the expected impact of the development of financial intermediation and the monetary policy transmission mechanism. Among other things, the negative impact of the crisis of confidence in the financial sector on the illegal activities of financial intermediaries is considered. It has been empirically confirmed that the interest rate, credit and foreign exchange channels of the monetary policy transmission mechanism can be used to overcome the crisis of erosion of the financial sector’s confidence in macroeconomic stability.

Aticle of Kuzmenko et al.(2020) [15] explains how to use data mining and bifurcation analysis based on limited information about a country’s overall performance to assess a country’s resilience to the involvement of its financial institutions in money laundering. Empirical calculations have shown that for the group of countries to which Ukraine belongs, the dynamic system is in a non-equilibrium state and is described as a "saddle" phase portrait. Therefore, the risk of using financial institutions for money laundering in Ukraine is high, although it is under certain state control.

Another group of authors (Lyeonov et al. (2020) [16] has developed a scientific and methodological approach to assessing the risk of financial monitoring in terms of the use of financial organizations for money laundering. This approach is based on the methods of multidimensional static analysis, descriptive, cluster and variance data analysis, gravity theory, non-linear econometric modeling, differential and bifurcation modeling, analysis of dynamic nonlinear systems. The study resulted in a developed model for a comprehensive assessment of the risks of financial organizations of countries in relation to money laundering, which provides for grouping countries according to the level of risk of money laundering, identifying whether the cluster belongs to the state; formation of an integrated index of both a rating assessment of the risk of money laundering and a risk assessment based on a gravity model; construction of a phase portrait of a dynamic risk system for the use of financial institutions of countries based on a non-linear econometric model.

An important area of research is research aimed at studying the growing role of fintech in payment relations and its impact on money laundering opportunities. The study by Petrushenko et al.(2018) [17] examines the cost and structure of investment flows as the most obvious indicators of fintech and describes the types of payment relations in it. The results of the study show the high potential of FinTech for processing cross-border payments and combating money laundering.

The authors (Paskevicius, A., & Keliuotyte-Staniuleniene, G. (2018) [18] paid considerable attention to the risky nature of financial innovations and the need to study their impact on capital markets, also from the point of view of the possibility of their use in illegal schemes. The authors have developed a panel model of the impact on capital markets in the countries of Central and Eastern Europe. The model proposed by the authors explains almost four-fifths of the changes in the capital market, expressed in terms of capitalization.

The authors Bilan, Y et al.(2019) [19] studied the relationship between the drivers of the shadow economy and the level of demand in the investment market. Based on the Shapiro-Wilk test, the normality of the distribution of capital investments and the level of the shadow economy of the EU countries and Ukraine were assessed. As a result, the following conclusions were obtained: the growth of the shadow economy, as well as the increase in the volume of money laundering operations, has a negative impact on the volume of capital investments in the country.

Based on the explication of structural and functional relationships, Brychko et al.2021) [20] developed a scientific and methodological approach to modeling the relationship between the illegal activities of financial intermediaries, a crisis of confidence in the financial sector and its deformations. Using structural equation modeling tools, a mediator analysis was carried out, the results of which confirmed the hypothesis that the crisis of confidence in the financial sectors leads to an increase in the number of shadow transactions involving financial intermediaries.

Also, the issues of financial monitoring are considered from the point of view of their impact on the financial security of the country. Vasilyeva et al.(2020) [21] developed a methodology for calculating a country’s financial sector governance quality index as a weighted average of a country’s overall indicator. compliance with the main international standards, rules and principles in the industry (including the rules and principles of financial monitoring). Analysis of the results shows that the level of economic development of countries does not play a key role in determining the level of financial security, and other determinants are more important, such as the rules and principles of financial monitoring.

Although there is a constantly growing number of scientific publications on financial monitoring, anti-money laundering, the shadow economy, and the impact of corruption on economic development, further research needs to determine the stability of the national financial monitoring system in dynamics. The dynamic stability of the national financial monitoring system subjects will allow to adequately assess the effectiveness of the existing national financial monitoring system in each country and determine the factors that have a significant impact on this stability.

## Materials and methods

### Study design

The study involves the following stages:

1. stage. Collection and systematization of statistical data for intellectual analysis of dynamic stability of national financial monitoring system subjects to actions on money laundering.
2. stage. Classification of input indicators into regressors and regressants and based on the selected regressors, the formation of an integrated indicator of the country’s financial system propensity to ALM. Among the many indicators of the statistical input base formed in the first stage, it is proposed to consider as regressors Index of economic freedom, Government Integrity, Monetary Sector credit to the private sector (% GDP). At the same time, regressants are Financial Freedom, Currency in Circulation (% GDP). The input indicators are distributed in such a way since the Financial Freedom indicator determines the banking system efficiency and indicates its government regulation independence, determining the state intervention degree in the financial sector. The index assesses the state regulation intensity of the financial service sector, the state intervention degree in banks and other financial institutions through direct or indirect ownership, the state’s influence on lending, the financial and capital market development degree, and openness to foreign competition. It means that the Financial Freedom indicator is a key indicator for assessing the effectiveness of the financial monitoring system of each country and can be related to the regressant.

The Currency in Circulation (% GDP) indicator is the currency that is physically used for transactions between consumers and businesses and is not stored in a bank, financial institution or central bank. Since most of the illegally obtained income is received in cash, this indicator indirectly describe the existing financial monitoring system, so it was also chosen as a regressant.

The Index of economic freedom, Government Integrity, Monetary Sector credit to the private sector (% GDP) were chosen as regressors because they describe the external conditions in which the national financial monitoring system operates: state regulation efficiency, the rule of law in the country, limited state power and openness of markets (Index of economic freedom), the level of perception of corruption in the public sector (Government Integrity) and the amount of financial support of the private sector to state and credit to state enterprises (Monetary Sector credit to the private sector) (% GDP) have the most significant impact on the effectiveness of the national financial monitoring system in the country and its dynamic stability.

Based on the selected regressors, the formation of the integrated indicator of the country’s financial system propensity to ALM involves a number of intermediate steps:

2.1. Normalization of regressors Financial Freedom (denote FF) and Currency in Circulation (% GDP) to bring to a comparable form via a nonlinear method using the logistics function:

$$\begin{array}{c} {ff}_{ij}^{n}=\frac{1}{e^{-k∙({ff}_{ij}-{ff}_{a})}}\\ {ff}_{a}=\frac{\underset{\mathrm{ij}}{\max}{ff}_{ij}+\underset{\mathrm{ij}}{\min}{ff}_{ij}}{2} \end{array}$$ (1)

where ${ff}_{ij}^{n}$–normalized value of the regression Financial Freedom in terms of the j-country for the j-year

*ff*_*ij*_–the actual value of the regression Financial Freedom in terms of the i-country for the j-year;

k–adjustment factor;

$\underset{\mathrm{ij}}{\max}{ff}_{ij}\;(\underset{\mathrm{ij}}{\min}{ff}_{ij})$–the maximum (respectively, minimum) value of the regression Financial Freedom on the set of values for the 15 considered countries from 2000 to 2021.

$$\begin{array}{c} {cc}_{ij}^{n}=\frac{1}{e^{-k∙({cc}_{ij}-{cc}_{a})}}\\ {cc}_{a}=\frac{\underset{\mathrm{ij}}{\max}\;{cc}_{ij}+\underset{\mathrm{ij}}{\min}\;{cc}_{ij}}{2} \end{array}$$ (2)

where ${cc}_{ij}^{n}$–normalized value of the regresant Currency in Circulation (% GDP) in terms of the i-country for the j-year;

cc_*ij*_–actual value of regresant Currency in Circulation (% GDP) in terms of the i-country for the j-year;

$\underset{\mathrm{ij}}{\max}\;{cc}_{ij}(\underset{\mathrm{ij}}{\min}\;{cc}_{ij})$–the maximum (respectively, minimum) value of the regresant Currency in Circulation (% GDP) on the set of values for the 15 considered countries from 2000 to 2020.

2.2. Calculation of the integrated indicator of the characteristic of the country’s financial system propensity to ALM based on the value inverse to the arithmetic mean of the normalized levels of regresant Financial Freedom and Currency in Circulation (% GDP):

$${PFSALM}_{ij}=1-\frac{{ff}_{ij}^{n}+{cc}_{ij}^{n}}{2}$$ (3)

where *PFSALM*_*ij*_—integrated indicator of the characteristics of the country’s financial system propensity to ALM in terms of the i-country for the j-year.

3 stage. Analysis of the dynamic stability of the national financial monitoring system to money laundering based on a binary approach. At this stage, the authors proposed to analyze the stability of the subjects of national financial monitoring systems in different countries to money laundering and study the stability dynamics. When exceeding the value of the integrated indicator of the characteristics of the country’s financial system propensity to the ALM level of 0.5, we can say about the instability, and otherwise—about the stability.


$$\left\{ \begin{array}{c} {If\;PFSALM}_{ij}\geq 0.5,unstable\\ {If\;PFSALM}_{ij}<0.5,stable \end{array}\right.$$ (4)

4 stage. Identification of relevant factors of dynamic stability/instability causality of national financial monitoring system subjects to money laundering based on the binary approach constructing a vector autoregression model (VAR) of dependence of the financial system propensity to ALM on regressors Index of economic freedom, Government Integrity, Monetary Sector credit to the private sector (% GDP) considering time lags for 15 considered countries. To implement this stage, authors proposed to use the EViews program Quick / Estimate VAR / VAR Type—Unrestricted VAR / Endogenous Variables—GI, IEF, MSCPS, PFSALM / Lag Interval for Endogenous—1 and 2. Vector autoregression model, the feasibility of which occurs when it is necessary to formalize interconnected time-series systems and analyze the dynamic effect of random perturbations on the system of variables, is as follows:

$$y_{t}=A_{1}∙y_{t-1}+A_{2}∙y_{t-2}+\ldots +A_{p}∙y_{t-p}+C∙x_{t}+\varepsilon_{t}$$ (5)

where $y_{t}-y_{t}={\left(y_{1t},y_{2t},\ldots ,y_{nt}\right)}^{Т}$ vector of endogenous dimensional variables *n*×1;

$x_{t}-x_{t}={\left(x_{1t},x_{2t},\ldots ,x_{kt}\right)}^{Т}$ vector of exogenous dimensional variables *k*×1;

A_1_, A_2_,…A_p_–constants, constant coefficients before lag endogenous variables;

*C* –dimension matrix *n*×*k* of coefficients before exogenous dimensional variables;

$\varepsilon_{t}-\varepsilon_{t}={\left(\varepsilon_{1t},\varepsilon_{2t},\ldots ,\varepsilon_{nt}\right)}^{Т}$ white noise, dimension vector *n*×1.

Considering an integral indicator of the country’s financial system propensity to ALM (PFSALM_t) as an endogenous variable with account of lag delays of one and two years, and Index of economic freedom, Government Integrity, Monetary Sector credit to private sector (% GDP) as exogenous variables with account of time lags in one and two years (respectively, GI_t, IEF_t, MSCPS_t), the vector autoregression (VAR) model (4) is as follows:

$${PFSALM}_{t}=a_{1}∙{GI}_{t-1}+a_{2}∙{GI}_{t-2}+a_{3}∙{IEF}_{t-1}+a_{4}∙{IEF}_{t-2}+a_{5}∙{MSCPS}_{t-1}+a_{6}∙{MSCPS}_{t-2}+a_{7}∙{PFSALM}_{t-1}+a_{8}∙{PFSALM}_{t-2}+\varepsilon_{t}$$ (6)

5 stage. Determination of specific features of dynamic stability/instability of national financial monitoring system subjects to money laundering based on the formalization of dependence of the country’s financial system propensity to ALM on regressors Index of economic freedom, Government Integrity, Monetary Sector credit to the private sector (% GDP) considering time lags for each of the 15 considered countries based on vector autoregression (VAR). In this stage, using vector autoregression tools, we will determine for each country which exogenous variables are significant and quantify their impact, as well as which lag delays explain the reflection of the country’s financial system propensity to ALM. Like the previous step, the EViews toolkit Quick / Estimate VAR / VAR Type—Unrestricted VAR / Endogenous Variables—GI, IEF, MSCPS, PFSALM / Lag Interval for Endogenous—1 and 2 is used to implement this step.

### Sampling and data collection

A statistical array of input indicators was created in the form of panel data for the period from 2000 to 2021 in terms of 15 European Union countries: Austria, Belgium, Cyprus, Estonia, Finland, France, Greece, Ireland, Italy, Latvia, Malta, Netherlands, Portugal, Slovak Republic, Spain. These countries to study the dynamic stability of the national financial monitoring system were selected due to the availability of the necessary statistics on the European Central Bank website for the selected time range in terms of 5 input indicators: Index of economic freedom, Government Integrity, Monetary Sector credit to the private sector (% GDP), Financial Freedom, Currency in Circulation (% GDP).

## Results

1 stage. Collection and systematization of statistical data to conduct an intellectual analysis of the dynamic stability of the national financial monitoring system to money laundering. A systematized statistics fragment is presented in Table 1.

**Table 1:** Fragment of static data of intellectual analysis of dynamic stability of national financial monitoring system subjects to money laundering.

Name	Country	Index of economic freedom	Government Integrity	Monetary Sector credit to private sector (% GDP)	Financial Freedom	Currency in Circulation (% GDP)
2000	Austria	68.4	75.0		70,00	
2001	Austria	68.1	76.0	89,71	70,00	4,83444
2002	Austria	67.4	77.0	88,52	70,00	5,37339
2003	Austria	67.6	78.0	87,81	70,00	5,86846
2004	Austria	67.6	78.0	87,77	70,00	6,25049
2005	Austria	68.8	80.0	94,32	70,00	6,13819
2006	Austria	71.1	84.0	94,44	70,00	6,91223
2007	Austria	71.6	87.0	92,92	70,00	7,18058
2008	Austria	71.4	86.0	95,81	70,00	6,89192
2009	Austria	71.2	81.0	97,74	70,00	8,01493
2010	Austria	71.6	81.0	98,53	70,00	7,74362
…	…	…	…	…	…	…
2012	Spain	69.1	61.0	158,18	80,00	10,26187
2013	Spain	68.0	62.0	146,52	70,00	10,92790
2014	Spain	67.2	62,60	130,62	70,00	10,73376
2015	Spain	67.6	59,00	119,26	70,00	11,88405
2016	Spain	68.5	60,00	111,81	70,00	11,36133
2017	Spain	63.6	57,20	105,90	70,00	12,98340
2018	Spain	65.1	51,50	99,49	70,00	11,74315
2019	Spain	65.7	51,90	94,65	70,00	11,88004
2020	Spain	66.9	55,10		70,00	14,47199
2021	Spain	69.9	70,30		70,00	

1 stage. Collection and systematization of statistical data to conduct an intellectual analysis of the dynamic stability of the national financial monitoring system to money laundering. The obtained results of normalization of regresants Financial Freedom and Currency in Circulation (% GDP) are systematized in Table 2.

**Table 2:** Normalized values of regresants Financial Freedom and Currency in Circulation (% GDP).

Country	Indicator	Years																				
		2000	2001	2002	2003	2004	2005	2006	2007	2008	2009	2010	2011	2012	2013	2014	2015	2016	2017	2018	2019	2020
Austria	FF	0,73	0,73	0,73	0,73	0,73	0,73	0,73	0,73	0,73	0,73	0,73	0,73	0,73	0,73	0,73	0,73	0,73	0,73	0,73	0,73	0,73
Austria	CC	0,22	0,32	0,33	0,34	0,35	0,35	0,37	0,37	0,36	0,39	0,38	0,37	0,39	0,40	0,38	0,40	0,39	0,42	0,40	0,42	0,46
Belgium	FF	0,73	0,73	0,73	0,73	0,73	0,73	0,73	0,88	0,88	0,88	0,73	0,73	0,73	0,73	0,73	0,73	0,73	0,73	0,73	0,73	0,73
Belgium	CC	0,22	0,29	0,33	0,34	0,35	0,35	0,37	0,37	0,37	0,40	0,39	0,38	0,39	0,40	0,39	0,41	0,40	0,43	0,41	0,42	0,46
Cyprus	FF	0,73	0,73	0,73	0,73	0,73	0,73	0,73	0,73	0,73	0,73	0,73	0,73	0,73	0,50	0,27	0,27	0,27	0,27	0,50	0,50	0,50
Cyprus	CC	0,22	0,22	0,22	0,22	0,22	0,37	0,38	0,36	0,37	0,41	0,40	0,39	0,42	0,45	0,47	0,50	0,47	0,50	0,46	0,48	0,53
Estonia	FF	0,73	0,73	0,95	0,95	0,95	0,95	0,95	0,95	0,88	0,88	0,88	0,88	0,88	0,88	0,88	0,88	0,88	0,88	0,88	0,73	0,73
Estonia	CC	0,22	0,22	0,22	0,22	0,35	0,34	0,34	0,32	0,30	0,31	0,27	0,48	0,50	0,50	0,48	0,52	0,50	0,53	0,48	0,49	0,54
Finland	FF	0,27	0,27	0,73	0,73	0,73	0,73	0,73	0,88	0,88	0,88	0,88	0,88	0,88	0,88	0,88	0,88	0,88	0,88	0,88	0,88	0,88
Finland	CC	0,22	0,26	0,31	0,33	0,34	0,34	0,36	0,36	0,35	0,39	0,38	0,37	0,39	0,40	0,38	0,41	0,40	0,43	0,40	0,42	0,45
France	FF	0,27	0,27	0,27	0,27	0,27	0,27	0,27	0,50	0,73	0,73	0,73	0,73	0,73	0,73	0,73	0,73	0,73	0,73	0,73	0,73	0,73
France	CC	0,22	0,27	0,33	0,34	0,35	0,35	0,37	0,37	0,37	0,40	0,39	0,38	0,40	0,41	0,39	0,42	0,41	0,45	0,43	0,44	0,49
Greece	FF	0,05	0,27	0,27	0,27	0,27	0,27	0,27	0,12	0,27	0,27	0,50	0,50	0,50	0,27	0,27	0,27	0,12	0,12	0,12	0,27	0,27
Greece	CC	0,22	0,34	0,35	0,36	0,37	0,37	0,38	0,39	0,38	0,43	0,44	0,46	0,51	0,55	0,54	0,60	0,60	0,66	0,62	0,59	0,68
Ireland	FF	0,73	0,95	0,95	0,95	0,95	0,95	0,95	0,95	0,95	0,95	0,88	0,73	0,73	0,73	0,73	0,73	0,73	0,73	0,73	0,73	0,73
Ireland	CC	0,22	0,30	0,29	0,29	0,31	0,30	0,31	0,32	0,32	0,39	0,39	0,38	0,40	0,40	0,38	0,35	0,35	0,36	0,34	0,34	0,35
Italy	FF	0,73	0,73	0,73	0,73	0,73	0,73	0,27	0,50	0,50	0,50	0,50	0,50	0,50	0,50	0,50	0,50	0,50	0,27	0,27	0,27	0,27
Italy	CC	0,22	0,32	0,33	0,35	0,35	0,35	0,38	0,38	0,38	0,42	0,41	0,40	0,43	0,45	0,43	0,46	0,45	0,49	0,46	0,48	0,54
Latvia	FF	0,73	0,73	0,73	0,73	0,73	0,73	0,73	0,73	0,73	0,50	0,27	0,27	0,27	0,27	0,27	0,27	0,50	0,50	0,50	0,50	0,50
Latvia	CC	0,22	0,22	0,22	0,22	0,22	0,22	0,22	0,22	0,22	0,22	0,37	0,38	0,39	0,30	0,57	0,60	0,58	0,62	0,57	0,58	0,63
Malta	FF	0,27	0,73	0,73	0,27	0,73	0,95	0,73	0,73	0,73	0,50	0,50	0,50	0,50	0,50	0,50	0,50	0,50	0,50	0,50	0,50	0,50
Malta	CC	0,22	0,22	0,22	0,22	0,22	0,74	0,74	0,50	0,47	0,49	0,46	0,45	0,47	0,47	0,43	0,43	0,42	0,44	0,41	0,44	0,48
Netherlands	FF	0,95	0,95	0,95	0,95	0,95	0,95	0,95	0,88	0,95	0,95	0,88	0,88	0,88	0,88	0,88	0,88	0,88	0,88	0,88	0,88	0,88
Netherlands	CC	0,22	0,27	0,31	0,32	0,33	0,33	0,34	0,35	0,34	0,37	0,37	0,36	0,38	0,39	0,37	0,40	0,39	0,42	0,39	0,41	0,43
Portugal	FF	0,27	0,27	0,27	0,27	0,27	0,27	0,27	0,27	0,27	0,50	0,50	0,50	0,50	0,50	0,50	0,50	0,50	0,50	0,50	0,50	0,50
Portugal	CC	0,32	0,31	0,36	0,38	0,40	0,40	0,42	0,43	0,42	0,48	0,47	0,47	0,52	0,53	0,50	0,54	0,52	0,57	0,53	0,53	0,58
Slovak Republic	FF	0,27	0,27	0,73	0,73	0,95	0,95	0,95	0,88	0,88	0,73	0,73	0,73	0,73	0,73	0,73	0,73	0,73	0,73	0,73	0,73	0,73
	CC	0,22	0,22	0,22	0,22	0,22	0,22	0,41	0,41	0,29	0,50	0,48	0,46	0,48	0,50	0,50	0,53	0,52	0,57	0,53	0,56	0,61
Spain	FF	0,73	0,73	0,73	0,73	0,73	0,73	0,73	0,88	0,88	0,88	0,88	0,88	0,88	0,73	0,73	0,73	0,73	0,73	0,73	0,73	0,73
Spain	CC	0,41	0,37	0,34	0,35	0,36	0,35	0,36	0,37	0,36	0,42	0,41	0,41	0,45	0,46	0,46	0,49	0,47	0,51	0,48	0,49	0,55

**Fig 1:**
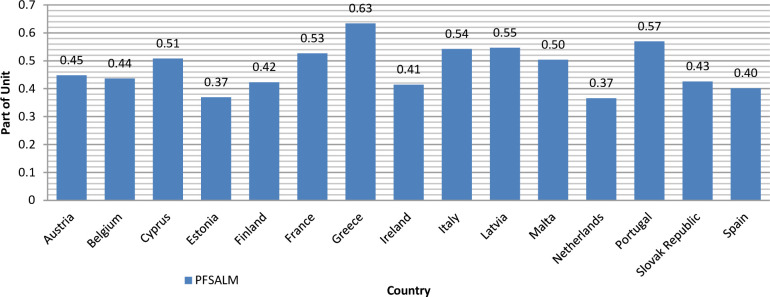
Dynamics of the average level of the integrated indicator in the characteristics of the country’s financial system propensity to ALM for the period from 2000 to 2020.

According to Formula (3), the calculation results will be presented in tabular form (Table 3) and give a visualization of the average level of each country for the studied time range (Fig 1).

**Table 3:** Dynamics of the integrated indicator of the characteristics of the country’s financial system propensity to ALM.

Country	Average value	Years																				
		2000	2001	2002	2003	2004	2005	2006	2007	2008	2009	2010	2011	2012	2013	2014	2015	2016	2017	2018	2019	2020
Austria	0,45	0,52	0,48	0,47	0,46	0,46	0,46	0,45	0,45	0,45	0,44	0,44	0,45	0,44	0,44	0,44	0,43	0,44	0,42	0,43	0,42	0,41
Belgium	0,44	0,52	0,49	0,47	0,46	0,46	0,46	0,45	0,37	0,38	0,36	0,44	0,45	0,44	0,43	0,44	0,43	0,44	0,42	0,43	0,42	0,40
Cyprus	0,51	0,52	0,52	0,52	0,52	0,52	0,45	0,44	0,46	0,45	0,43	0,44	0,44	0,43	0,53	0,63	0,62	0,63	0,62	0,52	0,51	0,49
Estonia	0,37	0,52	0,52	0,41	0,41	0,35	0,36	0,35	0,36	0,41	0,41	0,43	0,32	0,31	0,31	0,32	0,30	0,31	0,30	0,32	0,39	0,37
Finland	0,42	0,75	0,74	0,48	0,47	0,46	0,46	0,46	0,38	0,38	0,36	0,37	0,37	0,36	0,36	0,37	0,36	0,36	0,35	0,36	0,35	0,33
France	0,53	0,75	0,73	0,70	0,70	0,69	0,69	0,68	0,56	0,45	0,43	0,44	0,44	0,43	0,43	0,44	0,42	0,43	0,41	0,42	0,41	0,39
Greece	0,63	0,86	0,70	0,69	0,69	0,68	0,68	0,67	0,75	0,68	0,65	0,53	0,52	0,49	0,59	0,59	0,57	0,64	0,61	0,63	0,57	0,52
Ireland	0,41	0,52	0,38	0,38	0,38	0,37	0,37	0,37	0,37	0,36	0,33	0,37	0,45	0,44	0,43	0,44	0,46	0,46	0,45	0,47	0,46	0,46
Italy	0,54	0,52	0,47	0,47	0,46	0,46	0,46	0,68	0,56	0,56	0,54	0,54	0,55	0,53	0,53	0,54	0,52	0,53	0,62	0,63	0,63	0,60
Latvia	0,55	0,52	0,52	0,52	0,52	0,52	0,52	0,52	0,52	0,52	0,64	0,68	0,68	0,67	0,71	0,58	0,57	0,46	0,44	0,47	0,46	0,43
Malta	0,50	0,75	0,52	0,52	0,75	0,52	0,15	0,27	0,38	0,40	0,51	0,52	0,52	0,52	0,52	0,54	0,53	0,54	0,53	0,55	0,53	0,51
Netherlands	0,37	0,41	0,39	0,37	0,37	0,36	0,36	0,35	0,39	0,35	0,34	0,38	0,38	0,37	0,37	0,37	0,36	0,37	0,35	0,36	0,36	0,34
Portugal	0,57	0,71	0,71	0,69	0,68	0,67	0,67	0,65	0,65	0,65	0,51	0,52	0,52	0,49	0,48	0,50	0,48	0,49	0,47	0,49	0,49	0,46
Slovak Republic	0,43	0,75	0,75	0,52	0,52	0,41	0,41	0,32	0,36	0,41	0,38	0,39	0,40	0,39	0,39	0,38	0,37	0,37	0,35	0,37	0,35	0,33
Spain	0,40	0,43	0,45	0,46	0,46	0,46	0,46	0,45	0,37	0,38	0,35	0,35	0,35	0,34	0,40	0,41	0,39	0,40	0,38	0,39	0,39	0,36

Analysis of Fig 1 enables us to conclude that the countries with a high propensity of the financial system to ALM (the value of the integrated indicator is not less than 0.5 share of the unit) are Cyprus, France, Greece, Italy, Latvia, Malta, Portugal, which is 46,67% of the countries. For these countries, the variation in the country’s financial system propensity to ALM ranges from 0.50 to 0.63 units. It is due to the peculiarities of the economic and political components of these countries.

As for Cyprus, this country is an offshore zone, which encourages non-residents to invest in the country. Foreign investment in the Cyprus economy is often a way to "launder" money in other countries, which complicates the financial monitoring system work. In addition, Cyprus is a country that has problems with corruption in public authorities, which stimulates the growth of ALM-related transactions. In addition, establishing a strict financial monitoring system will reduce the number and volume of investment transactions by non-residents, which is contrary to Cyprus interests in terms of its economic development. It means that the country is not interested in building a stable financial monitoring system, which is reflected in the calculations of the integrated indicator of the country’s financial system propensity to ALM.

The territory ruled by Turkish Cypriots does not have the legal and institutional framework necessary for anti-money laundering. The casino and offshore banking sectors are key risks of money laundering. Due to international sanctions and the lack of TRNC recognition, the banking sector is largely isolated from international financial institutions. Banks operating in the area do not have access to the SWIFT system and have almost no correspondent banking relationships outside Turkey.

Greece has long-term economic problems, political and social instability, and a high share of the shadow economy. The high level of corruption in public authorities has a significant negative impact on the Greek economy. The above is the reason for the high country’s propensity to operations related to money laundering. Economic development, the population welfare increase, reducing corruption, and shadow economy are priorities for the Greek government. Solving these issues will lead in the short term to a change in the country’s financial system propensity for ALM.

Italy’s economy is the ninth largest in the world and the third-largest in the Eurozone. Italy has a complex anti-money laundering regime and legal framework. Still, it is characterized by the risk of money laundering arising from organized crime and a significant share of the country’s shadow economy. According to the National Institute of Statistics of Italy, the shadow sector in the country accounts for 12.1% of GDP, or about 235 billion dollars (€ 211 billion). In addition, tax crimes are widespread in the country (concealment of income to reduce or avoid tax payments). Tax crimes account for 75% of all income-generating crimes in Italy. Corruption is a big problem in Italy. The integrity of civil servants is undermined by their relationship with organized crime and business. All this significantly reduces the country’s financial monitoring system propensity to ALM.

Latvia is a regional financial center with many commercial banks and a significant deposit base of non-residents (accounting for more than half of the € 30 billion in the Latvian banking system). Cash from non-residents continues to flow across the border from neighboring Russia and other countries of the former Soviet Union. Latvia’s geographical location, large tax-free shadow economy (estimated at 25% of the total economy), and state corruption complicate the fight against money laundering in the country. The government identifies such primary sources of money laundering as tax evasion, organized criminal activity (prostitution and fraud committed by Russian and Latvian groups), and other forms of financial fraud. Corruption is a problem for businesses operating in Latvia. Demands for bribes and other irregular payments are widespread. Close relationships between civil servants and business, the influence of private interests related to the illegal financing of political parties, and the unethical behavior of companies increase the propensity of the financial monitoring system to ALM.

Malta’s location between North Africa and Italy makes it a transit center for drugs and human trafficking to Europe. The country’s offshore banking sector is relatively large (eight times its GDP), and its register of vessels is the largest in Europe. According to the Maltese police, the main sources of illegal income come from drug trafficking (including cocaine, heroin, and cannabis tar) and economic crimes, primarily fraud and misappropriation of public funds. Representatives of the financial sector emphasize the risks of attracting foreign deposits and investments of politically exposed persons (PEPs) from Eastern Europe and North Africa. Due to the above, Malta was forced in June 2021 to make a high-level political commitment to work with the FATF and MONEYVAL to increase the effectiveness of its AML / CFT regime.

Most of the money laundered in Portugal is related to drugs. In addition, significant revenues also come from corruption, trafficking in works of art and cultural artifacts, extortion, embezzlement, tax offenses, smuggling, prostitution, organized crime, gambling, and the facilitation of illegal immigration. Portugal has anti-money laundering laws and mechanisms that meet international standards and in 2017 took steps to improve money laundering legislation further.

Due to the large size of its economy, relative political stability, complex financial system, France is a place for money laundering. The sources of illegal income are state corruption, drugs and human trafficking, smuggling and other crimes related to organized crime. France has an informal economic sector, and underground money transfer and value transfer systems, such as hawala (used by immigrants), are accustomed to such systems in their countries. A significant share of money laundering is carried out with the help of virtual money. The use of virtual money is growing in France through online games and social networks. Sports teams have become another significant source of money laundering. Corruption is not a risk for businesses operating or planning to operate in France. The country’s investment climate is very favorable, and there is a strong legal framework to combat corruption.

We build a graph 2 to analyze the countries’ financial system propensity to ALM for the period from 2000 to 2020 in more detail.

**Fig 2:**
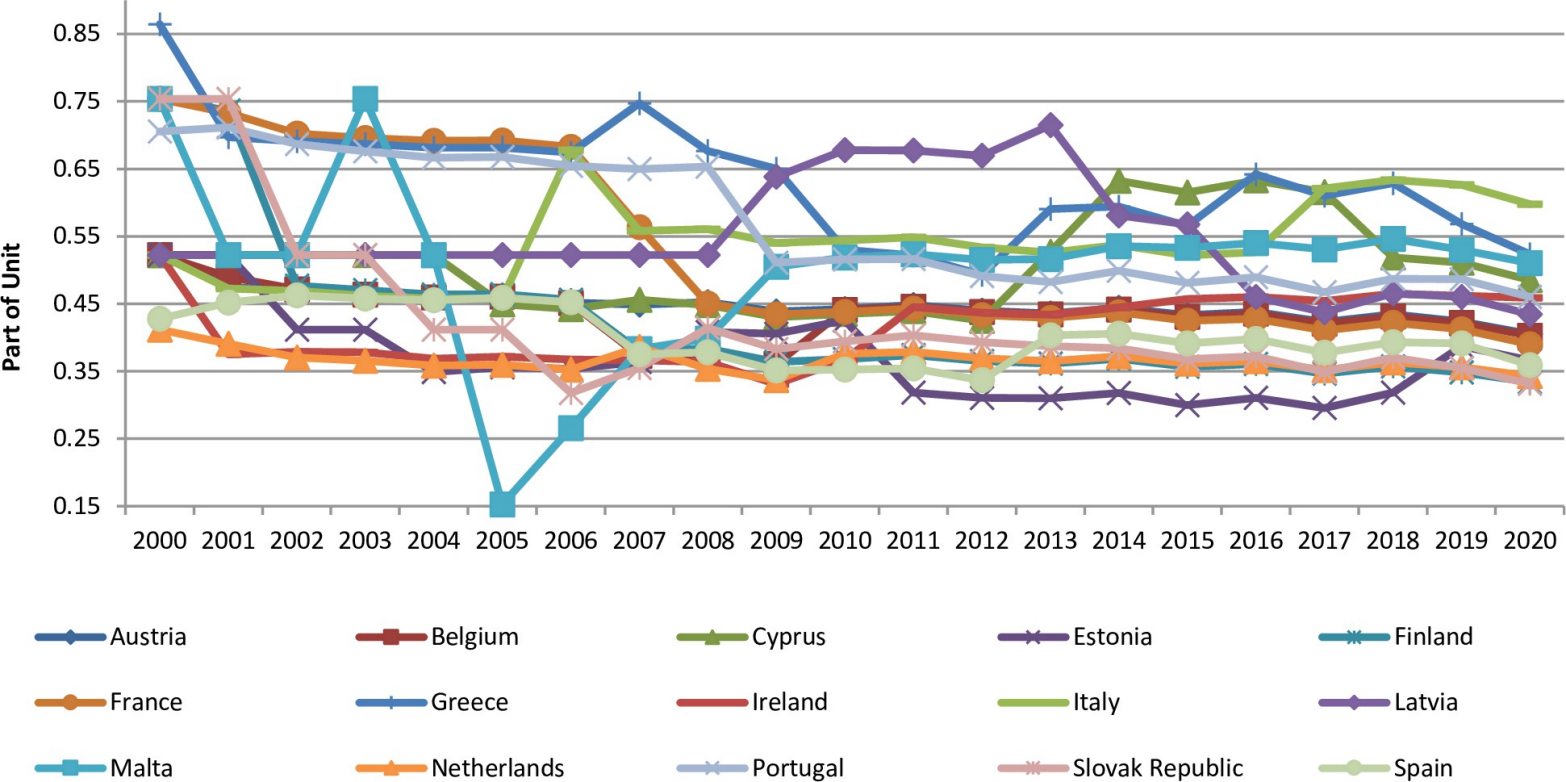
Comparability of the integrated indicator of the characteristics of the country’s financial system propensity to ALM for the period from 2000 to 2020 in terms of countries.

The data in Fig 2 show significant differences in the levels of the integrated indicator regarding the characteristics of the country’s financial system propensity to ALM for 2000–2020. The Netherlands, the Slovak Republic, Estonia, Spain, France and Belgium have the most robust financial monitoring systems for dealing with ALM. The lowest integrated indicator of the characteristic of the country’s financial system propensity to ALM is typical for Italy, Greece, Malta and Cyprus.

Therefore, we use the data in Table 3 (column "Average") and Figure to determine the countries for which the average study period from 2000 to 2020 is characterized by the stability of national financial monitoring systems of different countries to money laundering. Thus, the countries for which the stability of the national financial monitoring system subjects of different countries to money laundering, are Austria, Belgium, Estonia, Finland, France, Ireland, Netherlands, Slovak Republic, Spain.

3 stage. Analysis of the dynamic stability of the national financial monitoring system subjects to money laundering based on a binary approach. A similar approach is proposed to be used in the analysis of the dynamic stability of the national financial monitoring system subjects to money laundering based on the binary approach:

The calculations by Formula (4) are presented in Table 4.

**Table 4:** Analysis of the dynamic stability of the national financial monitoring system subjects to money laundering based on a binary approach.

Country	Years																					Total
	2000	2001	2002	2003	2004	2005	2006	2007	2008	2009	2010	2011	2012	2013	2014	2015	2016	2017	2018	2019	2020	
Austria	1	0	0	0	0	0	0	0	0	0	0	0	0	0	0	0	0	0	0	0	0	1
Belgium	1	0	0	0	0	0	0	0	0	0	0	0	0	0	0	0	0	0	0	0	0	1
Cyprus	1	1	1	1	1	0	0	0	0	0	0	0	0	1	1	1	1	1	1	1	0	12
Estonia	1	1	0	0	0	0	0	0	0	0	0	0	0	0	0	0	0	0	0	0	0	2
Finland	1	1	0	0	0	0	0	0	0	0	0	0	0	0	0	0	0	0	0	0	0	2
France	1	1	1	1	1	1	1	1	0	0	0	0	0	0	0	0	0	0	0	0	0	8
Greece	1	1	1	1	1	1	1	1	1	1	1	1	0	1	1	1	1	1	1	1	1	20
Ireland	1	0	0	0	0	0	0	0	0	0	0	0	0	0	0	0	0	0	0	0	0	1
Italy	1	0	0	0	0	0	1	1	1	1	1	1	1	1	1	1	1	1	1	1	1	16
Latvia	1	1	1	1	1	1	1	1	1	1	1	1	1	1	1	1	0	0	0	0	0	16
Malta	1	1	1	1	1	0	0	0	0	1	1	1	1	1	1	1	1	1	1	1	1	17
Netherlands	0	0	0	0	0	0	0	0	0	0	0	0	0	0	0	0	0	0	0	0	0	0
Portugal	1	1	1	1	1	1	1	1	1	1	1	1	0	0	0	0	0	0	0	0	0	12
Slovak Republic	1	1	1	1	0	0	0	0	0	0	0	0	0	0	0	0	0	0	0	0	0	4
Spain	0	0	0	0	0	0	0	0	0	0	0	0	0	0	0	0	0	0	0	0	0	0

The analysis of the dynamic stability of the national financial monitoring system subjects to the money laundering based on the binary approach allowed us to draw the following conclusions: for the whole period, only two countries had a stable financial monitoring system—the Netherlands and Spain. Austria and Belgium had only one period—in 2000, in which the sustainability of their financial monitoring systems was low. The most volatile financial monitoring systems for money laundering were France (8 periods), Cyprus and Portugal (12 periods each), Italy and Latvia (16 periods each), Malta (17 periods) and Greece (20 periods).

4 stage. Identification of relevant factors of dynamic stability / instability causality regarding the national financial monitoring system subjects to money laundering based on the binary approach constructing the vector autoregression model (VAR) of dependence of financial system propensity on Index of economic freedom, Government Integrity, Monetary Sector credit to private sector (% GDP) considering time lags for 15 observed countries.

Determining the coefficients of the vector autoregression model (VAR) of the dependence of the financial system of the country to ALM on the regression GI_t, IEF_t, MSCPS_t considering time lags, their standard errors and t-statistics of significance using EViews, we obtain the following results (Table 5).

**Table 5:** Vector autoregression estimates.

	GI	IEF	MSCPS	PFSALM
GI(-1)	0.971899	0.046996	0.078202	3.27E-05
	(0.05875)	(0.02048)	(0.09593)	(0.00053)
	[16.5417]	[2.29436]	[0.81523]	[0.06180]
GI(-2)	-0.004621	-0.037300	-0.072046	**-0.000616**
	(0.05857)	(0.02042)	(0.09562)	(0.00053)
	[-0.07890]	[-1.82686]	[-0.75347]	[-1.16848]
IEF(-1)	0.086672	0.904273	-0.072932	**-0.001997**
	(0.17654)	(0.06155)	(0.28823)	(0.00159)
	[0.49095]	[14.6928]	[-0.25303]	[-1.25703]
IEF(-2)	-0.051198	0.060806	0.050863	** 0.003076**
	(0.17851)	(0.06223)	(0.29146)	(0.00161)
	[-0.28680]	[0.97704]	[0.17451]	[1.91486]
MSCPS(-1)	0.020937	0.003869	1.592687	1.98E-06
	(0.02539)	(0.00885)	(0.04145)	(0.00023)
	[0.82472]	[0.43720]	[38.4260]	[0.00867]
MSCPS(-2)	-0.026693	-0.007654	-0.625140	5.60E-05
	(0.02517)	(0.00877)	(0.04109)	(0.00023)
	[-1.06055]	[-0.87233]	[-15.2132]	[0.24727]
PFSALM(-1)	-4.798004	-0.561652	-4.823649	** 0.927782**
	(6.50876)	(2.26911)	(10.6267)	(0.05857)
	[-0.73716]	[-0.24752]	[-0.45392]	[15.8393]
PFSALM(-2)	2.433461	1.210013	-0.465160	0.039683
	(6.50896)	(2.26918)	(10.6270)	(0.05858)
	[0.37386]	[0.53324]	[-0.04377]	[0.67745]
C	1.368914	1.819832	6.891387	-0.025273
	(4.11889)	(1.43594)	(6.72480)	(0.03707)
	[0.33235]	[1.26734]	[1.02477]	[-0.68181]
R-squared	0.943992	0.954360	0.977543	0.935332
Adj. R-squared	0.942398	0.953060	0.976904	0.933491
Sum sq. resids	3906.997	474.8520	10414.58	0.316423
S.E. equation	3.728794	1.299948	6.087904	0.033557
F-statistic	592.0197	734.4824	1528.999	508.0332
Log likelihood	-788.5855	-482.9949	-930.7490	577.4886
Akaike AIC	5.500589	3.393068	6.481027	-3.920611
Schwarz SC	5.614482	3.506961	6.594920	-3.806718
Mean dependent	66.65517	69.28138	95.78273	0.559727
S.D. dependent	15.53633	6.000075	40.05894	0.130119
Determinant resid covariance (dof adj.)		0.819882		
Determinant resid covariance		0.722744		
Log likelihood		-1598.887		
Akaike information criterion		11.27508		
Schwarz criterion		11.73066		

Note: Standard errors in () & t-statistics in []

Analysis of the data from the PFSALM column of Table 5 suggests that a statistically significant impact on the country’s financial system propensity to ALM have such exogenous variables as GI_(t-2)_, IEF_(t-1)_, IEF_(t-2)_, i.e., Government Integrity with a lag of 2 years and the Index of economic freedom considering the time delays of the regresant reflection under the influence of this regressor for 1 and 2 years. In addition to these exogenous variables, PFSALM_(t-1)_ has a significant impact on the country’s financial system propensity to ALM, i.e., the previous value. The statistical significance of these factors is confirmed by the calculated value of the Student’s criterion presented in Table 5 in the context of each coefficient in the linear regression Eq (5).

Given the numerical values of the coefficients, the vector autoregression (VAR) model of the dependence of the country’s financial system propensity to ALM on the regressors GI_t_, IEF_t_, MSCPS_t_ with time lags is as follows:

$$PFSALM=(3.26756e-05)∙{GI}_{t-1}-0.000616∙{GI}_{t-2}-0.001997∙{IEF}_{t-1}+0.00307∙{IEF}_{t-2}+(1.98179e-06)∙{MSCPS}_{t-1}+(5.60077e-05)∙{MSCPS}_{t-2}+0.927782∙{PFSALM}_{t-1}+0.0396831∙{PFSALM}_{t-2}-0.025273$$ (7)

**Fig 3:**
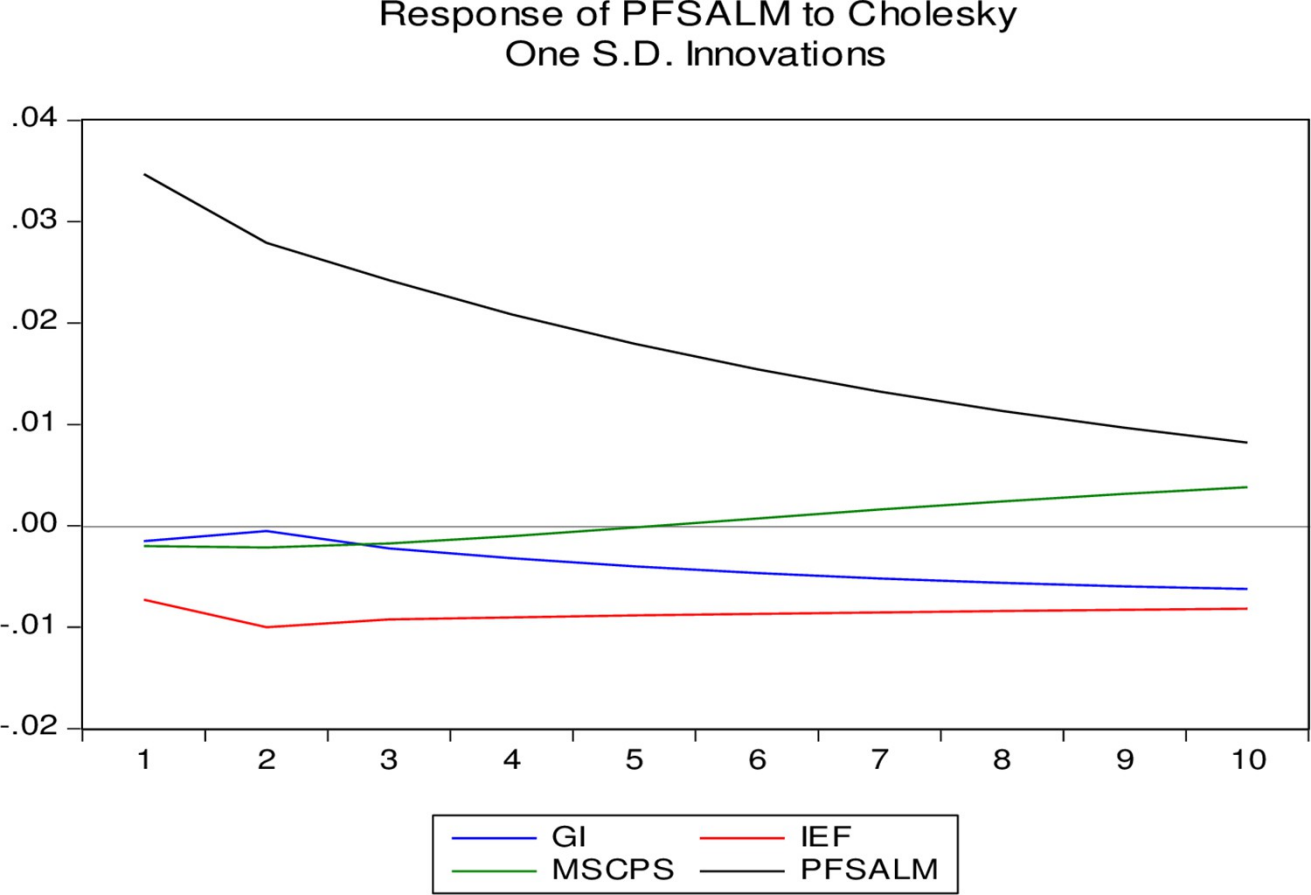
Visualization of the reflection of the country’s financial system propensity to ALM under the influence of regressors GI_t_, IEF_t_, MSCPS_t_ depending on the time lag.

Thus, the analysis of Eq (7) allows us to draw the following conclusions in terms of statistically confirmed trends: the current value of the financial system propensity to ALM by 92.78% is determined by its previous value; the Government Integrity indicator acts as a de-stimulator for the PFSALM performance indicator, i.e., when the level of Government Integrity increases by 1%, the country’s financial system propensity to ALM will decrease by 0.000616 shares per unit with a lag of two years; Index of economic freedom has a specific nature of the impact on the performance indicator—when the value of this indicator increases by 1% for lag delay in one year, the value of PFSALM will decrease by 0.001997 units, and for lag delay in two years will change the trend and increase by 0.003076 shares per unit, respectively. It indicates the nature of this indicator as a stimulant, but with the existing inflection point of reflection. Fig 3 confirms this trend.

Analyzing the visualization of the country’s financial system propensity to ALM under the influence of regressors GIt, IEFt, MSCPSt presented in Fig 3 depending on the time lag; we can state the following: with increasing time lag, the influence of its previous values of PFSALM gradually decreases; with increasing lag delay, the impact of the indicator Monetary Sector credit to the private sector (% GDP) on the resultant feature gradually increases, but with a probability of 0.95 it is not statistically confirmed; in the short term (with a lag of one year) indicators GIt, IEFt act as a stimulator and destimulator, respectively, for the country’s financial system propensity to ALM, for further time lags, starting from 2 years, trends for these indicators change dramatically to the opposite.

**Fig 4:**
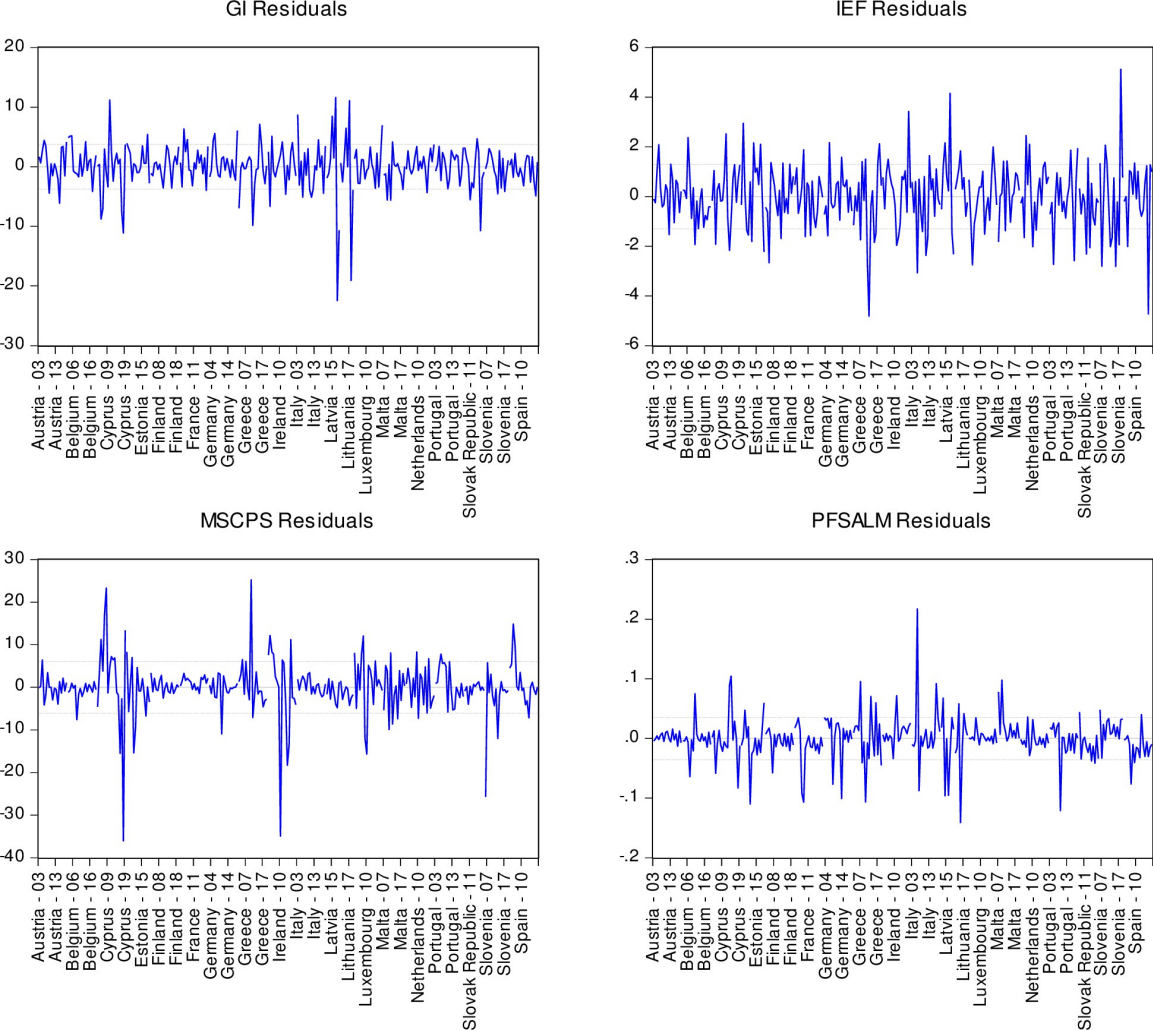
Distribution of the country’s financial system propensity balances to ALM, of regressors Index of economic freedom, Government Integrity, Monetary Sector credit to private sector (% GDP).

It is important for further use of the constructed vector autoregression model (VAR) results of the dependence of the country’s financial system propensity to ALM on regressors Index of economic freedom, Government Integrity, Monetary Sector credit to private sector (% GDP) to prove the accuracy and adequacy to state the values given in Table 5: R-squared at the level of 0.9353, i.e., the variation of the effective feature of PFSALM by 93.53% is explained by the variation of factor GI_t_, IEF_t_, MSCPS_t_ considering the time lags of reflection; F-statistic at the level of 508.03, which is significantly higher than the critically acceptable level, and indicates the statistical significance of the obtained model (7); Akaike information criterion (11.28) and Schwarz criterion (11.73), which indicate a fairly good fit of statistics to the model; distribution of residuals for both regression and regressors (Fig 4).

The obtained results are systematized in tabular form (Table 6).

**Table 6:** Vector autoregression estimates.

	PFSALM	PFSALM	PFSALM	PFSALM	PFSALM	PFSALM	PFSALM	PFSALM
	Austria	Belgium	Cyprus	Estonia	Finland	France	Greece	Ireland
GI(-1)	5.65E-05	0.000638	0.004734	0.004220	-0.000385	-0.006810	-0.002407	0.003271
	(0.00075)	(0.00364)	(0.00440)	(0.00638)	(0.00349)	(0.00369)	(0.00524)	(0.00200)
	[0.07536]	[0.17548]	[1.07487]	[0.66108]	[-0.11025]	[-1.84654]	[-0.45980]	[1.63872]
GI(-2)	0.000719	-0.003335	-0.001314	0.003726	0.000145	-0.005014	-0.002820	0.000828
	(0.00085)	(0.00240)	(0.00383)	(0.00847)	(0.00333)	(0.00342)	(0.00506)	(0.00181)
	[0.84678]	[-1.39095]	[-0.34293]	[0.44013]	[0.04357]	[-1.46708]	[-0.55697]	[0.45726]
IEF(-1)	-0.001440	-0.007813	-0.025314	0.007269	-0.004605	-0.000369	-0.018789	-0.001471
	(0.00281)	(0.00628)	(0.02097)	(0.00891)	(0.00809)	(0.00798)	(0.01123)	(0.00382)
	[-0.51195]	[-1.24391]	[-1.20721]	[0.81585]	[-0.56913]	[-0.04627]	[-1.67366]	[-0.38550]
IEF(-2)	-0.003553	-0.000621	0.015397	-0.009360	0.010524	-0.003863	0.002680	-0.005749
	(0.00233)	(0.00755)	(0.02185)	(0.01989)	(0.00801)	(0.00847)	(0.00741)	(0.00602)
	[-1.52436]	[-0.08231]	[0.70479]	[-0.47055]	[1.31396]	[-0.45582]	[0.36143]	[-0.95579]
MSCPS(-1)	-0.000591	-0.000639	-0.001137	0.003549	-0.005005	-0.002919	0.001440	-0.001424
	(0.00088)	(0.00438)	(0.00104)	(0.00217)	(0.00462)	(0.00353)	(0.00274)	(0.00042)
	[-0.67403]	[-0.14585]	[-1.09702]	[1.63297]	[-1.08231]	[-0.82775]	[0.52620]	[-3.38847]
MSCPS(-2)	0.000733	-0.003146	0.001358	-0.004624	0.002978	-0.009013	-0.003482	0.001166
	(0.00076)	(0.00464)	(0.00117)	(0.00363)	(0.00394)	(0.00543)	(0.00250)	(0.00038)
	[0.96750]	[-0.67744]	[1.15660]	[-1.27284]	[0.75622]	[-1.65913]	[-1.39423]	[3.06521]
PFSALM(-1)	-0.212797	-0.087484	0.497074	0.580349	0.293588	0.164283	0.157634	0.071502
	(0.30363)	(0.32980)	(0.57333)	(0.84337)	(0.46855)	(0.35593)	(0.33464)	(0.30613)
	[-0.70085]	[-0.26526]	[0.86700]	[0.68813]	[0.62659]	[0.46157]	[0.47106]	[0.23357]
PFSALM(-2)	0.363512	-0.108417	-0.176545	0.457266	0.141163	-0.311606	0.403917	-0.047945
	(0.29461)	(0.30503)	(0.45034)	(0.58523)	(0.16635)	(0.29701)	(0.44918)	(0.33391)
	[1.23386]	[-0.35543]	[-0.39203]	[0.78134]	[0.84861]	[-1.04915]	[0.89923]	[-0.14359]
C	0.653299	1.523398	0.791542	-0.295793	-0.023503	2.735708	1.597071	0.691092
	(0.37993)	(0.54955)	(2.67818)	(0.90372)	(1.35445)	(0.76360)	(0.61508)	(0.56856)
	[1.71951]	[2.77207]	[0.29555]	[-0.32731]	[-0.01735]	[3.58264]	[2.59654]	[1.21551]
R-squared	0.868002	0.752176	0.830119	0.768069	0.913797	0.979090	0.760286	0.934204
F-statistic	6.575861	3.035123	4.886482	2.069771	10.60055	46.82515	3.171645	14.19845
Akaike AIC	-7.045885	-4.521473	-3.093927	-3.604539	-4.832001	-4.368160	-2.929851	-5.030452
Schwarz SC	-6.604772	-4.080361	-2.652815	-3.193716	-4.390888	-3.927047	-2.488738	-4.589339
	PFSALM	PFSALM	PFSALM	PFSALM	PFSALM	PFSALM		PFSALM
	Italy	Latvia	Malta	Netherlands	Portugal	Slovak Republic		Spain
GI(-1)	0.000896	0.001836	0.009100	0.001407	-0.006925	-0.000699		-0.000189
	(0.00784)	(0.00257)	(0.00695)	(0.00216)	(0.00552)	(0.00049)		(0.00503)
	[0.11436]	[0.71497]	[1.31003]	[0.65170]	[-1.25554]	[-1.41678]		[-0.03754]
GI(-2)	0.000444	0.001129	-0.001670	0.001794	0.000148	-1.60E-05		0.000406
	(0.00758)	(0.00501)	(0.00212)	(0.00219)	(0.00574)	(0.00077)		(0.00439)
	[0.05865]	[0.22528]	[-0.78901]	[0.81945]	[0.02570]	[-0.02088]		[0.09246]
IEF(-1)	0.030836	0.002391	0.006787	0.001091	-0.003155	0.009353		7.49E-05
	(0.02575)	(0.01591)	(0.01162)	(0.00355)	(0.01073)	(0.00322)		(0.00654)
	[1.19762]	[0.15026]	[0.58399]	[0.30714]	[-0.29395]	[2.90858]		[0.01144]
IEF(-2)	0.013819	0.006496	-0.001369	-0.002872	-0.005807	0.004201		0.010227
	(0.02746)	(0.01834)	(0.01388)	(0.00323)	(0.01023)	(0.00296)		(0.00752)
	[0.50316]	[0.35410]	[-0.09863]	[-0.88884]	[-0.56769]	[1.41935]		[1.36021]
MSCPS(-1)	-0.006412	-0.002225	0.004277	-8.03E-05	-0.004423	-0.005064		-0.000760
	(0.01059)	(0.00377)	(0.00241)	(0.00113)	(0.00273)	(0.00137)		(0.00133)
	[-0.60545]	[-0.59083]	[1.77343]	[-0.07133]	[-1.61919]	[-3.69149]		[-0.56986]
MSCPS(-2)	0.011610	0.005397	-0.004413	0.001762	0.004174	0.004707		-0.000106
	(0.00977)	(0.00404)	(0.00243)	(0.00130)	(0.00297)	(0.00134)		(0.00142)
	[1.18809]	[1.33625]	[-1.81738]	[1.35481]	[1.40455]	[3.52293]		[-0.07485]
PFSALM(-1)	0.324963	0.538334	0.410129	-0.247120	0.501151	-0.625538		0.397570
	(0.34737)	(0.33048)	(0.42438)	(0.28628)	(0.29570)	(0.13211)		(0.40756)
	[0.93549]	[1.62895]	[0.96643]	[-0.86321]	[1.69480]	[-4.73515]		[0.97549]
PFSALM(-2)	0.097402	0.242120	0.629759	-0.361859	0.825162	0.067340		0.091902
	(0.35663)	(0.36110)	(0.27331)	(0.26677)	(0.39750)	(0.07415)		(0.44241)
	[0.27312]	[0.67051]	[2.30423]	[-1.35646]	[2.07589]	[0.90820]		[0.20773]
C	-2.929223	-0.871021	-0.748631	0.247175	0.817181	-0.267648		-0.399508
	(2.51439)	(1.79355)	(1.77579)	(0.42726)	(0.75813)	(0.38033)		(0.71240)
	[-1.16498]	[-0.48564]	[-0.42158]	[0.57851]	[1.07790]	[-0.70372]		[-0.56079]
R-squared	0.459917	0.838112	0.945691	0.520799	0.905987	0.982328		0.794934
F-statistic	0.851566	5.824247	8.706500	1.086808	9.636870	20.84468		3.876475
Akaike AIC	-2.337655	-2.965258	-4.668220	-5.808951	-3.486648	-7.687374		-4.211487
Schwarz SC	-1.896542	-2.520073	-4.277101	-5.367838	-3.045535	-7.323694		-3.770374

Based on the data in Table 6, we write auto regression models of the dependence of the country’s financial system propensity to ALM on regressors Index of economic freedom, Government Integrity, Monetary Sector credit to private sector (% GDP) considering time lags:

–Austria:

$$PFSALM=(5.651056e-05)∙{GI}_{t-1}+0.000719∙{GI}_{t-2}-0.001440∙{IEF}_{t-1}-0.003552∙{IEF}_{t-2}-0.000591∙{MSCPS}_{t-1}+0.000733∙{MSCPS}_{t-2}-0.212796∙{PFSALM}_{t-1}+0.3635116∙{PFSALM}_{t-2}+0.653299$$ (8)

–Belgium:-

$$PFSALM=0.000638∙{GI}_{t-1}-0.003335∙{GI}_{t-2}-0.007812∙{IEF}_{t-1}-0.000621∙{IEF}_{t-2}-0.000639∙{MSCPS}_{t-1}-0.003145∙{MSCPS}_{t-2}-0.087483∙{PFSALM}_{t-1}-0.108417∙{PFSALM}_{t-2}+1.523398$$ (9)

–Cyprus:

$$PFSALM=0.004733∙{GI}_{t-1}-0.001314∙{GI}_{t-2}-0.025313∙{IEF}_{t-1}+0.0153970108373∙{IEF}_{t-2}-0.001136∙{MSCPS}_{t-1}+0.001357∙{MSCPS}_{t-2}+0.497073∙{PFSALM}_{t-1}-0.176545∙{PFSALM}_{t-2}+0.791541$$ (10)

–Estonia:

$$PFSALM=0.004219∙{GI}_{t-1}+0.003726∙{GI}_{t-2}+0.007268∙{IEF}_{t-1}-0.009360∙{IEF}_{t-2}+0.003549∙{MSCPS}_{t-1}-0.004624∙{MSCPS}_{t-2}+0.580348∙{PFSALM}_{t-1}+0.457266∙{PFSALM}_{t-2}-0.295792$$ (11)

–Finland:

$$PFSALM=-0.000385∙{GI}_{t-1}+0.000145∙{GI}_{t-2}-0.004604∙{IEF}_{t-1}+0.010524∙{IEF}_{t-2}-0.005004∙{MSCPS}_{t-1}+0.002977∙{MSCPS}_{t-2}+0.293587∙{PFSALM}_{t-1}+0.141162∙{PFSALM}_{t-2}-0.023503$$ (12)

–France:

$$PFSALM=-0.00681∙{GI}_{t-1}-0.005013∙{GI}_{t-2}-0.000369∙{IEF}_{t-1}-0.003862∙{IEF}_{t-2}-0.002918∙{MSCPS}_{t-1}-0.009013∙{MSCPS}_{t-2}+0.164283∙{PFSALM}_{t-1}-0.311605∙{PFSALM}_{t-2}+2.735707$$ (13)

–Greece:

$$PFSALM=-0.002407∙{GI}_{t-1}-0.002820∙{GI}_{t-2}-0.018789∙{IEF}_{t-1}+0.002679∙{IEF}_{t-2}+0.001439∙{MSCPS}_{t-1}-0.003481∙{MSCPS}_{t-2}+0.157633∙{PFSALM}_{t-1}+0.403917∙{PFSALM}_{t-2}+1.597070$$ (14)

–Ireland:

$$PFSALM=0.003270∙{GI}_{t-1}+0.000827∙{GI}_{t-2}-0.001471∙{IEF}_{t-1}-0.005749∙{IEF}_{t-2}-0.001423∙{MSCPS}_{t-1}+0.001165∙{MSCPS}_{t-2}+0.071502∙{PFSALM}_{t-1}-0.047944∙{PFSALM}_{t-2}+0.691092$$ (15)

–Italy:

$$PFSALM=0.000896∙{GI}_{t-1}+0.000444∙{GI}_{t-2}+0.030836∙{IEF}_{t-1}+0.013818∙{IEF}_{t-2}-0.006411∙{MSCPS}_{t-1}+0.011610∙{MSCPS}_{t-2}+0.324963∙{PFSALM}_{t-1}+0.097402∙{PFSALM}_{t-2}-2.929222$$ (16)

–Latvia:

$$PFSALM=0.001836∙{GI}_{t-1}+0.001129∙{GI}_{t-2}+0.002391∙{IEF}_{t-1}+0.006496∙{IEF}_{t-2}-0.002225∙{MSCPS}_{t-1}+0.005397∙{MSCPS}_{t-2}+0.538334∙{PFSALM}_{t-1}+0.242120∙{PFSALM}_{t-2}-0.871021$$ (17)

–Malta:

$$PFSALM=0.0091∙{GI}_{t-1}-0.001669∙{GI}_{t-2}+0.006787∙{IEF}_{t-1}-0.001369∙{IEF}_{t-2}+0.004277∙{MSCPS}_{t-1}-0.004413∙{MSCPS}_{t-2}+0.410129∙{PFSALM}_{t-1}+0.629758∙{PFSALM}_{t-2}-0.74863$$ (18)

–Netherlands:

$$PFSALM=0.001407∙{GI}_{t-1}+0.001793∙{GI}_{t-2}+0.00109∙{IEF}_{t-1}-0.002872∙{IEF}_{t-2}-(8.031951e-05)∙{MSCPS}_{t-1}+0.001762∙{MSCPS}_{t-2}-0.247119∙{PFSALM}_{t-1}-0.361858∙{PFSALM}_{t-2}+0.247174$$ (19)

–Portugal:

$$PFSALM=-0.006924∙{GI}_{t-1}+0.000147∙{GI}_{t-2}-0.003155∙{IEF}_{t-1}-0.005807∙{IEF}_{t-2}-0.004423∙{MSCPS}_{t-1}+0.004173∙{MSCPS}_{t-2}+0.50115∙{PFSALM}_{t-1}+0.825161∙{PFSALM}_{t-2}+0.81718$$ (20)

–Slovak Republic:

$$PFSALM=-0.000699∙{GI}_{t-1}-(1.60076e-05)∙{GI}_{t-2}+0.009352∙{IEF}_{t-1}+0.004201∙{IEF}_{t-2}-0.005064∙{MSCPS}_{t-1}+0.004706∙{MSCPS}_{t-2}-0.625538∙{PFSALM}_{t-1}+0.067339∙{PFSALM}_{t-2}-0.267647$$ (21)

–Spain:

$$PFSALM=-0.000188∙{GI}_{t-1}+0.000405∙{GI}_{t-2}+(7.486275e-05)∙{IEF}_{t-1}+0.010227∙{IEF}_{t-2}-0.000759∙{MSCPS}_{t-1}-0.000105∙{MSCPS}_{t-2}+0.397569∙{PFSALM}_{t-1}+0.091901∙{PFSALM}_{t-2}-0.399508$$ (22)

Stage 5. Determination of specific features of dynamic stability / instability of national financial monitoring system subjects to money laundering based on dependence of country’s financial system propensity to ALM on regressors Index of economic freedom, Government Integrity, Monetary Sector credit to private sector (% GDP) considering time lags for each of the 15 countries based on autoregression vector (VAR). We will define for each country which exogenous variables are significant and quantify their impact, as well as which lag delays explain the reflection of the country’s financial system propensity to ALM, based on Tables 6 and 7.

**Table 7:** Statistically significant coefficients before the influential indicators of the country’s financial system propensity to ALM.

	Austria	Belgium	Cyprus	Estonia	Finland	France	Greece	Ireland
GI(-1)			0.004734			-0.006810		0.003271
GI(-2)		-0.003335				-0.005014		
IEF(-1)		-0.007813	-0.025314				-0.018789	
IEF(-2)	-0.003553				0.010524			
MSCPS(-1)			-0.001137	0.003549	-0.005005			-0.001424
MSCPS(-2)			0.001358	-0.004624		-0.009013	-0.003482	0.001166
PFSALM(-2)	0.363512					-0.311606		
	Italy	Latvia	Malta	Netherlands	Portugal	Slovak Republic		Spain
GI(-1)			0.009100		-0.006925	-0.000699		
IEF(-1)	0.030836					0.009353		
IEF(-2)						0.004201		0.010227
MSCPS(-1)			0.004277		-0.004423	-0.005064		
MSCPS(-2)	0.011610	0.005397	-0.004413	0.001762	0.004174	0.004707		
PFSALM(-1)		0.538334			0.501151	-0.625538		
PFSALM(-2)			0.629759	-0.361859	0.825162			0.091902

The analysis of Table 7 allows us to conclude that for each country, different exogenous variables are significant. At the same time, despite it, we can identify some common trends. In particular, the influential exogenous variable for most studied countries (for eleven of the fifteen countries) was the Monetary Sector credit to the private sector (% GDP) with a lag of 2 years (MSCPS (-2)). It means that for eleven countries, changes in the volume of MSCPS lead to a change in the stability of the financial monitoring system in 2 years.

For seven countries from the selected list, the exogenous variable MSCPS (-1) has a significant impact, i.e., the change in the volume of MSCPS leads to a change in the financial monitoring system stability in one year.

Exogenous variables GI (-1) and PFSALM (-2) were influential for the six countries on the list. It means that a change in the value of the Government Integrity indicator for a particular country leads to a change in the stability of its financial monitoring system with a lag of one year. The variable PFSALM (-2) means a significant impact of the stability indicator in the financial monitoring system for the period *t*−2 (with a lag of 2 years) on the current stability indicator.

For nine of the fifteen surveyed countries, only two of these factors affect the financial monitoring system stability, and these factors are different for each country. Such countries include Austria (0.41), Belgium (0.4), Estonia (0.37), Finland (0.33), Greece (0.52), Italy (0.6), Latvia (0,43), the Netherlands (0.34) and Spain (0.36). Nine of these countries are characterized by a high financial monitoring system stability, except for Greece and Italy.

The largest number of exogenous variables that have a significant impact on the financial monitoring system stability is peculiar for Portugal (0.46) (five factors) and Slovak Republic (0.33) (6 factors).

Thus, we can conclude that each country has its own unique set of exogenous factors that significantly impact the financial monitoring system stability. Their identification is an important aspect since it will allow public authorities to understand the influential mechanism of certain decisions on the existing financial monitoring system stability in the country, and thus will increase its efficiency.

## Conclusion

As a result of the study, authors proposed a method to assess the integrated indicator of the characteristics of the country’s financial system propensity to ALM. It allowed to adequately assess the existing financial monitoring systems of selected countries of the European Union. The list of fifteen selected countries was divided into countries characterized by the national financial monitoring system stability to money laundering (Austria, Belgium, Estonia, Finland, France, Ireland, Netherlands, Slovak Republic, Spain) and countries for which such stability is uncharacteristic (Malta, Greece, Cyprus, Portugal, Italy, Latvia). Similar data were obtained after analyzing the dynamic stability of the national financial monitoring system subjects to money laundering using a binary approach.

Determining the coefficients of the vector autoregression model (VAR) of the dependence of the country’s financial system propensity to ALM on regressions GI_t, IEF_t, MSCPS_t with time lags allowed to state that statistically significant influence on the country’s financial system propensity to ALM is exerted by exogenous variables such as GI_(t-2), IEF_ (t-1), IEF_ (t-2), i.e., Government Integrity with a lag of 2 years and the Index of economic freedom with the time delays of the regression’s reflection under the influence of this regressor for 1 and 2 years. In addition to these exogenous variables, a significant impact on the country’s financial system propensity to ALM has and PFSALM_ (t-1), i.e., the previous value. The calculated value of the Student’s criterion confirmed the statistical significance of these factors.

In addition, vector autoregression models (VAR) of the dependence of the country’s financial systems propensity to ALM on the regressors GI_t, IEF_t, MSCPS_t, were built, with time lags in general and for each studied country separately. The general vector autoregression (VAR) model shows that the current value of the country’s financial system propensity to ALM by 92.78% is determined by its previous value. The Government Integrity indicator acts as a destimulator for the PFSALM performance indicator, i.e., when the level of Government Integrity increases by 1%, the country’s financial system propensity to ALM will decrease by 0.000616 shares per unit with a lag of two years. The impact of indicator—Index of economic freedom on the performance is specific—when this indicator increases by 1% for lag delay in one year, the value of PFSALM will decrease by 0.001997 units, and for lag delay in two years will change the trend and increase by 0.003076 shares per unit, respectively. It generally indicates the nature of this indicator as a stimulator but with the existing inflection point of reflection. The accuracy and adequacy of the obtained model was confirmed by the following: R-squared at the level of 0.9353, i.e., the effective feature variation of PFSALM by 93.53% is explained by the variation of factor features GI_t, IEF_t, MSCPS_t with time lags of reflection; F-statistic at the level of 508.03, that is significantly higher than the critically acceptable level and indicates the statistical significance of the obtained model (7); Akaike information criterion (11.28) and Schwarz criterion (11.73), which indicate a fairly good fit of statistics to the model; distribution of residuals for both regressor and regressors.

Significant exogenous variables and their quantitative impact on the integrated indicator of the characteristic of the country’s financial system propensity to ALM for each of the studied countries were also identified. Since each country has its unique set of exogenous factors that have a significant impact on the sustainability of its financial monitoring system, identifying and assessing their impact will help governments to consider such exogenous factors in anti-money laundering policies and ensure its effectiveness growth in the future.

The article was prepared based on the results of a research funded by the National Research Fund of Ukraine "Optimization and automation of financial monitoring processes to increase information security in Ukraine." (registration number: 0120U104810).
